# Recent Developments of Advanced Broadband Photodetectors Based on 2D Materials

**DOI:** 10.3390/nano15060431

**Published:** 2025-03-11

**Authors:** Yan Tian, Hao Liu, Jing Li, Baodan Liu, Fei Liu

**Affiliations:** 1 School of Materials Science and Engineering, Northeastern University, No. 11, Wenhua Road, Shenyang 110819, China; tiany29@mail2.sysu.edu.cn (Y.T.); lijing1@mail.neu.edu.cn (J.L.); 2 State Key Laboratory of Optoelectronic Materials and Technologies, Guangdong Province Key Laboratory of Display Material and Technology, School of Electronics and Information Technology, Sun Yat-sen University, Guangzhou 510275, China; liuh378@mail2.sysu.edu.cn; 3 Foshan Graduate School of Innovation, Northeastern University, No. 2, Zhihui Road, Shunde District, Foshan 528300, China

**Keywords:** 2D materials, broadband photodetection, enhancing photodetection performance, photoresponse mechanism

## Abstract

With the rapid development of high-speed imaging, aerospace, and telecommunications, high-performance photodetectors across a broadband spectrum are urgently demanded. Due to abundant surface configurations and exceptional electronic properties, two-dimensional (2D) materials are considered as ideal candidates for broadband photodetection applications. However, broadband photodetectors with both high responsivity and fast response time remain a challenging issue for all the researchers. This review paper is organized as follows. Introduction introduces the fundamental properties and broadband photodetection performances of transition metal dichalcogenides (TMDCs), perovskites, topological insulators, graphene, and black phosphorus (BP). This section provides an in-depth analysis of their unique optoelectronic properties and probes the intrinsic physical mechanism of broadband detection. In Two-Dimensional Material-Based Broadband Photodetectors, some innovative strategies are given to expand the detection wavelength range of 2D material-based photodetectors and enhance their overall performances. Among them, chemical doping, defect engineering, constructing heterostructures, and strain engineering methods are found to be more effective for improving their photodetection performances. The last section addresses the challenges and future prospects of 2D material-based broadband photodetectors. Furthermore, to meet the practical requirements for very large-scale integration (VLSI) applications, their work reliability, production cost and compatibility with planar technology should be paid much attention.

## 1. Introduction

With the rapid advancement of information technology, there is an increasing demand for photodetectors capable of covering a wide spectral range, from visible to infrared light and even terahertz [1,2,3]. These detectors play an important role in aerospace exploration, remote sensing [4,5,6,7], environmental monitoring [8,9,10,11], and high-speed imaging [12,13,14,15]. Over the past decades, the narrow-bandgap semiconductors, such as mercury cadmium telluride (HgCdTe), indium antimonide (InSb) and silicon, have been intensively researched due to their intrinsic bandgaps that make them suitable for broadband photodetection applications [16,17,18]. Although traditional photodetectors have made some progress, they still encounter some limitations [19,20]. For instance, HgCdTe photodetectors require a low operation temperature below 70 K to achieve high-performance detection. Moreover, they are not friendly to the environment due to the existence of heavy metal elements Hg and Cd, and their fabrication process is complicated and expensive.

Two-dimensional (2D) materials such as transition metal dichalcogenides (TMDCs) [21,22,23,24], perovskite [25,26,27], graphene [28,29,30], topological insulators [31,32,33], black phosphorus (BP) [34,35] and MXenes [36,37,38] attract tremendous interest owing to their distinctive physical and chemical properties. Unlike traditional thin films that require strict lattice matching to avoid defects and strain, 2D layered materials can be seamlessly integrated with various substrates without such constraints [39]. This flexibility not only simplifies the fabrication process but also enhances device performance by reducing defects and improving carrier mobility. Additionally, 2D materials exhibit tunable bandgaps [40,41], strong light-matter interactions [42], high carrier mobility [43], and exceptional mechanical flexibility [44,45], making them highly suitable for broadband photodetection [46,47]. For example, Hu et al. [48] demonstrated an ultra-broadband photoresponse (375 nm–10 μm) through the integration of ferroelectric and low-dimensional semiconductor materials. Using the electric field reversible modulation characteristics of bipolar 2D van der Waals (vdW) heterojunctions, Zhai et al. [49] implemented wide-spectrum (365–980 nm) convolution processing and recognition within sensors. Zhang et al. [50] designed an innovative 2D vertical heterostructure photodetector with an exceptionally high optical responsivity and detectivity over a broad wavelength range (405–1550 nm). In addition to 2D materials, engineered semiconductor nanostructures have emerged as a promising platform for broadband photodetectors. These nanostructures, such as quantum dots, nanowires, and nanoribbons, offer unique advantages due to their tunable bandgaps, enhanced light absorption, and efficient charge transport properties. However, engineered semiconductor nanostructures also face several challenges that need to be addressed for practical applications including high fabrication costs, environmental instability, scalability issues, performance limitations, and potential health and environmental concerns. Given the unique advantages of 2D materials, our review focuses on these materials to provide an in-depth exploration of their potential in broadband photodetection.

Despite there being some research progress with 2D material-based photodetectors, the fabrication of high-performance broadband photodetectors with self-powered, rapid switching speed and high responsivity is still challenging due to the limitation of photosensitive materials themselves, such as low carrier mobility, high dark currents, low light absorption efficiency, and poor environmental stability. Additionally, the photoresponse speed and detection spectral range of these photodetectors are often affected by carrier recombination, trap states, or material crystallinity. Therefore, modulation of the optoelectronic properties of 2D materials and the fabrication of a high-performance photodetector are highly essential for their actual application in broadband photodetection.

The purpose of this review is to provide a comprehensive perspective, which analyzes and summarizes recent research strategies for enhancing the work performance of 2D material-based devices for broadband photodetection. First, the optoelectronic properties of pristine 2D materials are introduced to explore the intrinsic broadband photodetection mechanism. Next, the strategies for enhancing the work performance of 2D material-based photodetectors will be discussed in detail. Finally, we will address the challenges of realizing high-performance broadband photodetectors and their potential applications in future.

## 2. Two-Dimensional Material-Based Broadband Photodetectors

There are several key mechanisms within photodetectors, including the photovoltaic effect, photoconductive effect, photogating effect, photothermoelectric effect, and bolometric effect [51]. In the photovoltaic effect, charges are generated when photons with energy higher than the material’s bandgap are absorbed. These charges are then separated by the built-in electric field at the p–n junction or Schottky junction. The photoconductive effect occurs when high-energy photons create additional free carriers, increasing the electrical conductivity of the semiconductor material. These carriers are further separated by applied bias voltages, resulting in a net photocurrent. Additionally, the photogating effect is a particular case of the photoconductive effect, where photo-generated carriers modulate the device’s electrical properties without significant current flow. The photothermoelectric effect involves local light illumination, which increases the material temperature via the Seebeck effect, generating a detectable current due to the resulting temperature gradient. The bolometric effect is based on the resistivity change of a temperature-sensitive material due to uniform light heating, which alters the current under an external bias. In this article, we focus on photodetectors primarily based on the photoconductive effect and photothermoelectric effect, which are widely used in broadband detection applications. Devices predominantly governed by the photothermoelectric effect can achieve an exceedingly broad detection spectrum, extending from the visible to the near-infrared, mid-infrared, far-infrared regions, and even terahertz waves. In contrast, devices of photoconductive effect are restricted to a more defined wavelength range, depending on their bandgap width.

In this section, the properties of five commonly used 2D materials are explored for realizing broadband photodetection: transition metal dichalcogenides (TMDCs), graphene, black phosphorus (BP), topological insulators, and perovskites [52,53,54].

### 2.1. Transition Metal Dichalcogenides

Transition Metal Dichalcogenides (TMDCs) are layered materials consisting of a layer of transition metal atoms (molybdenum (Mo) or tungsten (W)) sandwiched between two layers of chalcogen atoms (sulfur (S), selenium (Se), or tellurium (Te)). These materials are characterized by the chemical formula MX_2_, where M represents the transition metal and X denotes the chalcogen. A key feature of TMDCs is their bandgap, which is significantly influenced by the number of layers, thereby conferring upon them a broad spectral response capability. The sensitivity of TMDCs to layer thickness endows them with the ability to absorb a broad spectrum of light, rendering them highly desirable for applications in broadband photodetection.

**Figure 1:**
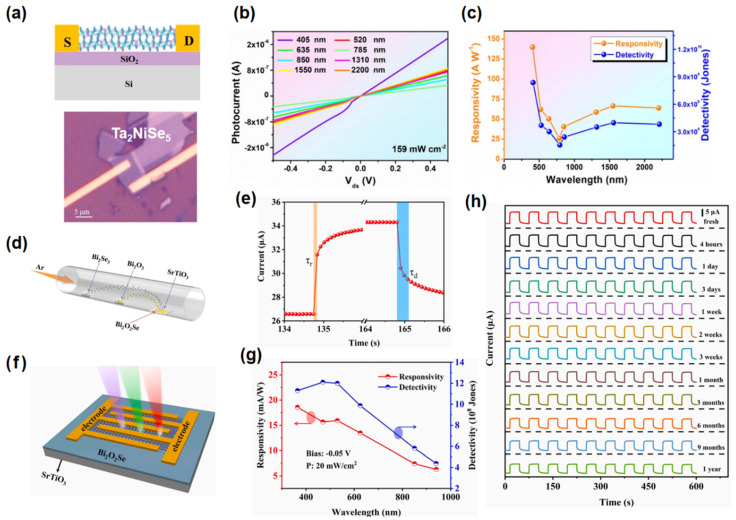
(**a**) Schematic illustration of the multilayer Ta_2_NiSe_5_ photodetector. (**b**) Photocurrent curves versus V_ds_ under the different illumination of lasers. (**c**) Responsivity and Detectivity of the Ta_2_NiSe_5_ photodetector as a function of wavelength. Reproduced with permission from [56], 2D Mater.; published by IOP, 2023. (**d**) Schematic illustration of the CVD process for growing 2D Bi_2_O_2_Se film on SrTiO_3_ substrate. (**e**) The magnified rising and falling edges at 850 nm. (**f**) Schematic diagram of the Bi_2_O_2_Se photodetector. (**g**) R and D* of the Bi_2_O_2_Se photodetector as a function of wavelength. (**h**) Time-resolved current of the fresh Bi_2_O_2_Se photodetector and stored in air atmosphere for 4 h, 1 day, 3 days, 1 week, 2 weeks, 3 weeks, 1 month, 3 months, 6 months, 9 months and 1 year. Reproduced with permission from [57], Chem. Eng. J.; published by Elsevier, 2023.

For instance, Pargam et al. [55] developed a 2D SnSe thin film photodetector capable of detecting light from 532 to 1064 nm owing to its narrow bandgap, maintaining stable performance even after 30 days of air exposure. Guo et al. [56] fabricated a high-performance photodetector using high-quality ternary Ta_2_NiSe_5_ nanosheets (Figure 1a), featuring a narrow bandgap of 0.25 eV. As illustrated in Figure 1b, the device exhibits broadband photodetection capabilities across the visible to infrared (IR) spectral (405–2200 nm) at room temperature. At 405 nm, it achieves a maximum responsivity of up to 280 A/W. Notably, at 2200 nm, it exhibits an impressive responsivity of 63.9 A/W and a detectivity of 3.8 × 10^9^ Jones, as shown in Figure 1c. Ren et al. [57] synthesized a high-quality 2D Bi_2_O_2_Se thin film on a SrTiO_3_ substrate via chemical vapor deposition (Figure 1d), exhibiting a perfect atomic arrangement and a well-defined interface with the substrate. As illustrated in Figure 1e–g, a photodetector based on the 2D Bi_2_O_2_Se thin film was developed to detect across ultraviolet, visible, and infrared wavelengths from 365 to 940 nm. It demonstrates a rapid response time of 32 ms and 44 ms, a peak response sensitivity of 136 mA/W, and a detectivity of up to 2 × 10^9^ Jones. Moreover, the Bi_2_O_2_Se photodetector showed remarkable stability performance in an ambient atmosphere after one year of storage, as demonstrated in Figure 1h. Transition metal dichalcogenides (TMDCs) have shown remarkable potential for broadband photodetection, but these materials also face challenges related to carrier mobility and environmental stability. Additionally, the synthesis of high-quality TMDCs with uniform thickness and few defects is still a challenge. Moreover, the scalability of TMDC-based devices for large-scale production needs further research.

### 2.2. Two-Dimensional Perovskite

Beyond conventional 2D materials such as TMDCs, perovskites have recently emerged as promising candidates for broadband photodetectors [58,59]. Perovskite materials have a crystal structure of ABX_3_, where A is typically an organic molecule or alkali metal ion, B is a transition metal ion, and X is a halogen ion. They have attracted significant interest in broadband photodetection due to their remarkable optical properties, including tunable bandgap [60,61] and excellent charge carrier performance [62].

**Figure 2:**
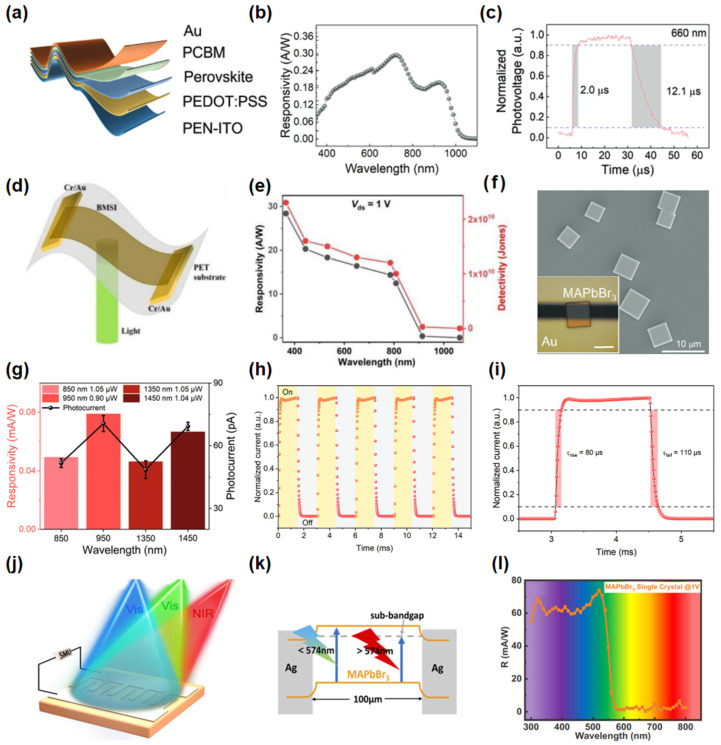
(**a**) Structure of a flexible Sn-Pb perovskite photodetector. (**b**) R of the Sn-Pb photodetector as a function of wavelength. (**c**) Response speed curve. Reproduced with permission from [63], Adv. Opt. Mater.; published by Wiley, 2024. (**d**) Schematic device structure of the (BA)_2_(MA)Sn_2_I_7_ photodetector. (**e**) Photocurrent dependence on light wavelength measured at 1 V bias voltage. Reproduced with permission from [64], Opt. Express; published by OSA, 2023. (**f**) SEM images of MAPbBr_3_ nanoplates. The inset in (**f**) shows an optical image of the as-prepared photodetector. (**g**) Responsivity and photocurrent of MAPbBr_3_ photodetector under infrared laser irradiation. (**h**) Normalized time-resolved photocurrent of MAPbBr_3_ photodetector under pulsed laser irradiation with frequency of 333 Hz. (**i**) A single normalized cycle in (**k**). Reproduced with permission from [65], Small; published by Wiley, 2023. (**j**) Schematic diagram of the MAPbBr_3_/Ag photodetector. (**k**) Energy levels of the MAPbBr_3_ photodetector under different illumination conditions. (**l**) R of the MAPbBr_3_ photodetector at different wavelengths. Reproduced with permission from [66], Opt. Express; published by OSA, 2022.

Li et al. [63] synthesized a stable 2D Cs_0.05_MA_0.45_FA_0.5_Sn_0.5_Pb_0.5_I_3_(Sn-Pb) (which MA  =  CH_3_NH_3_^+^, FA = CH(NH_2_)_2_^+^) perovskite, with reduced Sn vacancy density and suppressed yellow phase impurities through Sn^2+^ enrichment Cs^+^ ions incorporation. As given in Figure 2a, the photodetector based on Sn-Pb perovskite exhibits a response range spanning from ultraviolet to near-infrared wavelengths (Figure 2b). Under irradiation with a 720 nm laser, the device demonstrated a responsivity of 0.29 A/W and achieved ultrafast response characteristics with rise and fall times of 2 μs and 12.1 μs, as shown in Figure 2c. Xu et al. [64] presented a novel flexible optoelectronic detector utilizing (BA)_2_(MA)Sn_2_I_7_ (which BA  =  C_4_H_12_N^+^, MA  =  CH_3_NH_3_^+^), which achieved a wide-spectral response across the ultraviolet-visible-near-infrared spectrum from 365 to 1064 nm, as illustrated in Figure 2d,e. The device exhibited high responsivities of 28.4 A/W at 365 nm and 0.02 A/W at 1064 nm, corresponding to detectivities of 2.3 × 10^10^ and 1.8 × 10^7^ Jones, respectively. Furthermore, it demonstrated stable photodetection performance after 1000 bending cycles. Figure 2f shows that Mei et al. [65] employed a low-temperature vapor-diffusion method to synthesize a 2D MAPbBr_3_ nanoplates photodetector. The high specific surface area and surface trap-assisted absorption characteristics of the nanoplates contribute to the device’s outstanding performance in the near-infrared spectral range from 850 to 1450 nm, as depicted in Figure 2g. Notably, it achieves an external quantum efficiency (EQE) of 1200% and a detectivity of 5.37 × 10^12^ Jones, while also demonstrating a rapid response rise/fall time of 80/110 μs, as shown in Figure 2h,i. Xu et al. [66] investigated a broadband photodetector based on MAPbBr_3_ (Figure 2j), capable of spanning the ultraviolet to near-infrared spectral range. Figure 2k presents a schematic diagram of the photo-responsive mechanism. The mechanism for photocurrent generation in the visible light spectrum was elucidated as the photoelectric effect. For incident light wavelengths shorter than the cutoff wavelength of 574 nm, single-photon absorption serves as the primary absorption mechanism. In contrast, for wavelengths exceeding 574 nm, sub-gap trap state absorption predominates. In the near-infrared region, thermal effects govern the photoresponse mechanism. Figure 2l illustrates the device’s optical response across various wavelength bands, revealing that it attains a high plateau prior to reaching a wavelength of 520 nm. Despite significant advancements in the development of 2D perovskites, these materials still face challenges, particularly in terms of environmental stability and reproducibility. The long-term operation stability of 2D perovskite devices is another critical issue, especially when exposed to varying conditions such as humidity, temperature, and light. Moreover, the cost-effectiveness of the synthesis process is crucial for large-scale applications.

### 2.3. Graphene

Graphene, a 2D material composed of carbon atoms arranged in an sp^2^ hybridized configuration, forms a planar hexagonal lattice structure [67]. It exhibits zero bandgap semi-metallic characteristics, which means that the energy required for electrons to transition from the valence band to the conduction band is minimal. This allows graphene to absorb a wide range of photon energies across the spectrum [68]. Additionally, the linear dispersion of Dirac electrons in graphene suggests that for any excitation, there is always a resonance of an electron–hole pair, contributing to its high bandwidth photodetection capability. Combined with graphene’s exceptionally high electron mobility, these properties enable it to rapidly generate photocurrents in response to light, making it an ideal material for broadband photodetection.

**Figure 3:**
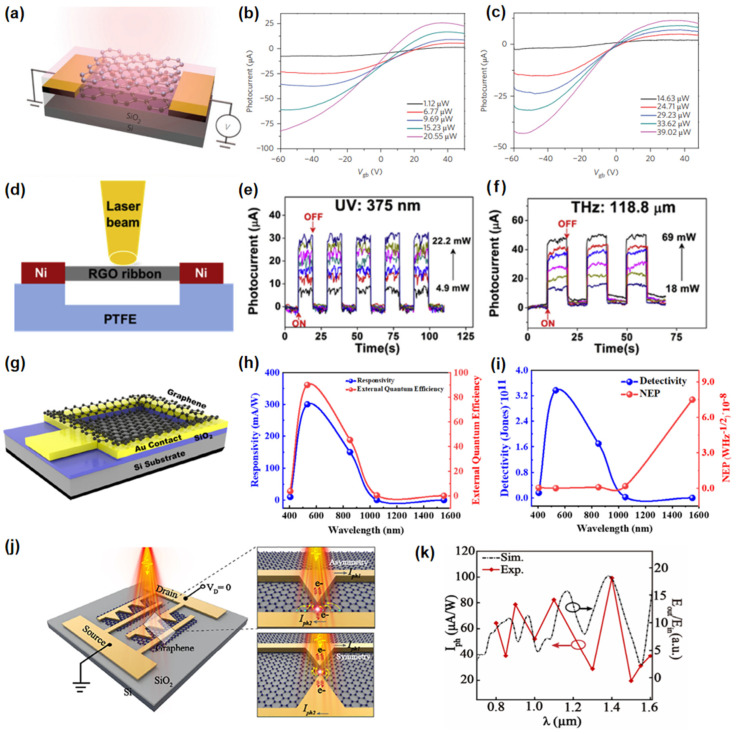
(**a**) Structure of the graphene photodetector. (**b**) Gate dependence of a photocurrent under different illumination powers with excitation wavelengths at 1.3 mm (**c**) Gate dependence of a photocurrent under different illumination powers with excitation wavelengths at 3.2 mm. Reproduced with permission from [69], Nat. Nanotechnol.; published by Macmillan, 2014. (**d**) Schematic diagram of the fully suspended rGO thin film photodetector. (**e**,**f**) Performance of a fully suspended rGO thin film photodetector from the UV to THz spectral region. Reproduced with permission from [70], Carbon; published by Elsevier, 2014. (**g**) Schematic diagram of the graphene photodetector device. (**h**) Responsivity and external quantum efficiency of the device. (**i**) Specific detectivity and noise equivalent power of the device. Reproduced with permission from [71], Energy Technol.; published by Wiley, 2023. (**j**) Schematic diagram of the graphene photodetectors. (**k**) Normalized photocurrent profile acquired upon various excitation light wavelengths. Reproduced with permission from [72], Nano Lett.; published by American Chemical Society, 2024.

Liu et al. [69] reported an ultra-broadband photodetector based on a graphene bilayer heterostructure, with the device structure illustrated in Figure 3a. Under illumination, the top layer generates hot carriers that tunnel into the bottom layer, resulting in charge accumulation at the gate and producing a pronounced optoelectronic gating effect on channel conductivity. This device demonstrates room-temperature light detection capability across a spectrum from visible to mid-infrared wavelengths, achieving a mid-infrared responsivity exceeding 1 A/W, as shown in Figure 3b,c. However, due to the inherently low optical absorption of graphene, the responsivity of graphene-based photodetectors is relatively low. Yang et al. [70] developed a novel method for the preparation of highly conductive reduced graphene oxide (rGO) and constructed a fully suspended photodetector as illustrated in Figure 3d. This detector displayed the fastest and broadest optical response among all reported rGO photodetectors. As demonstrated in Figure 3e,f, the response time is approximately 100 ms, with a response range spanning from ultraviolet to terahertz spectral region. Qasim et al. [71] established a high-performance, self-powered broadband photodetector, as illustrated in Figure 3g. This device is constructed from a stack formed on an n-Si substrate via graphene. As illustrated in Figure 3h, the device exhibits a remarkable broadband spectral response ranging from visible light (405 nm) to infrared (1,550 nm). This phenomenon can be attributed to the formation of a Schottky barrier at the interface between graphene and silicon. Under illumination, photons with energies below the silicon bandgap are absorbed, generating photo-induced charge carriers that are subsequently separated by the built-in electric field, resulting in a photogenerated current. Under illumination at 532 nm and with a rapid rise time of 320 µs, the device achieves a responsivity of 300 mA/W, a detectivity of 3.37 × 10^11^ Jones, and an external quantum efficiency of 90%, as depicted in Figure 3i. Our team has proposed an asymmetric plasmonic nanostructure array on planar graphene [72], as illustrated in Figure 3j. Under excitation, the non-centrosymmetric metallic nanostructures exhibit a strong light-matter interaction with the local field near the tip surface, resulting in an asymmetric electric field. These characteristics can enhance the generation of hot electrons within graphene, leading to directed diffusion currents. As shown in Figure 3k, the device demonstrates significant optical responsiveness across a wavelength range of 0.8 to 1.6 µm. Under laser excitation at 1.4 µm and zero bias, it achieves a responsivity of 25 mA/W and a noise equivalent power of about 0.44 nW/Hz^1/2^. Graphene-based broad photodetectors have demonstrated high responsivity and fast response times owing to their large carrier mobility. However, the low optical absorption of graphene limits its overall performance. Moreover, the large-scale production of high-quality graphene with consistent properties is still a challenge.

### 2.4. Topological Insulators

Topological insulators (TIs), with their bulk exhibiting narrow bandgaps and their surfaces featuring zero bandgaps, possess surface states that connect the conduction and valence bands of the bulk, enabling broad spectral range detection [73]. The unique electronic structure of topological insulators, characterized by their robust topologically protected surface states, is the cornerstone of their potential for wide-spectrum photodetection. These surface states are impervious to backscattering and non-magnetic impurities, thereby ensuring efficient charge transport and light absorption across a diverse spectral of wavelengths.

**Figure 4:**
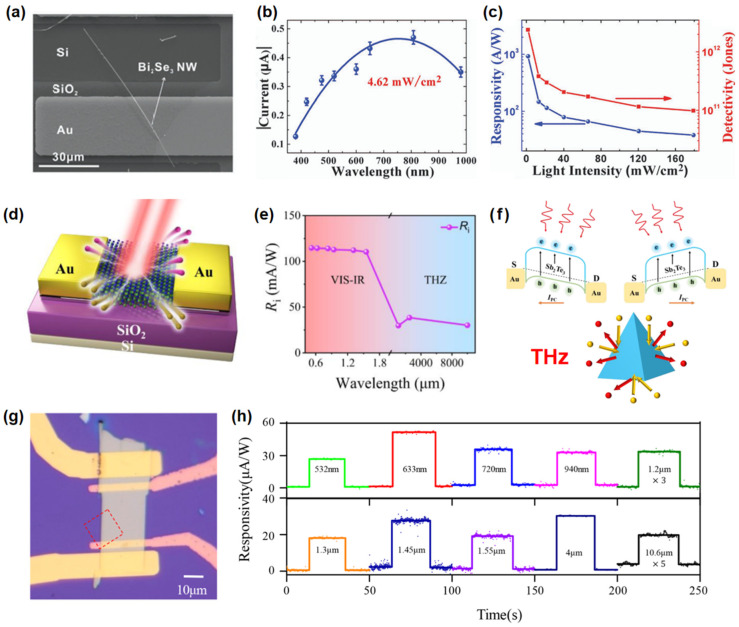
(**a**) Typical SEM image of the Bi_2_Se_3_ nanowire device. (**b**) Spectral response of the device measured in the wavelength range of 380–980 nm. (**c**) Responsivity and detectivity of the device as a function of light intensity. Reproduced with permission from [74], J. Mater. Chem. C; published by Royal Society of Chemistry, 2016. (**d**) Schematic illustration of the Sb_2_Te_3_ photodetector structure. (**e**) Wavelength dependency of current responsivity, at zero bias and room temperature, covering both the visible to IR and THz spectral bands. (**f**) The top half panel demonstrates the band diagrams of the Sb_2_Te_3_ photodetector under illumination with limited positive (**left**) and negative (**right**) bias voltages, the bottom half panel demonstrates asymmetry in elastic scattering due to the wedge effect. Reproduced with permission from [75], AIP Adv.; published by AIP, 2024. (**g**) Optical image of a TaIrTe_4_ device. (**h**) Broadband photoresponse of TaIrTe_4_ photodetector. Photoresponse of the TaIrTe_4_ device for different excitation wavelengths at 298 K. Reproduced with permission from [76], ACS Nano; published by American Chemical Society, 2018.

Liu et al. [74] constructed a Bi_2_Se_3_ nanowire/Si photodetector, as illustrated in Figure 4a, showing exceptional photoelectric detection performance. Due to its small bulk bandgap, the device covers a wide spectral range for photodetection, spanning from 380 to 980 nm as detailed in Figure 4b. Moreover, an effective Schottky barrier is established at the interface. When subjected to varying optical power excitation at a wavelength of 808 nm, the device consistently shows favorable photocurrent responses, achieving a peak responsivity of 10^3^ A/W and a swift response time of around 45 ms, as given in Figure 4c. Chen et al. [75] introduced a super-broadband photodetector that integrates dual mechanisms based on the topological insulator Sb_2_Te_3_, as revealed in Figure 4d. The response range of this photodetector spans from 520 nm to 0.28 THz (as shown in Figure 4e). Upon irradiation with a 520 nm laser, the device demonstrates a room-temperature responsivity of 114.6 mA/W and a detectivity of 1.78 × 10^8^ cm^2^ Hz^1/2^ W^−1^. At a frequency of 0.12 THz, the room-temperature responsivity is 38.5 mA/W, with a detectivity of 3.44 × 10^10^ cm^2^ Hz^1/2^ W^−1^, and an observed response time of 20 ps. As presented in Figure 4f, the device’s operation in the visible-to-infrared spectrum is primarily due to the photoconductive effect, while its terahertz functionality is largely attributed to the asymmetric scattering behavior of topological surface states. Lai et al. [76] reported a wide-spectrum photodetector based on TaIrTe_4_, with its device structure shown in Figure 4g. The photodetector demonstrated a broadband response from 532 nm to 10.6 μm, as seen in Figure 4h, suggesting that its detection range can be extended into the far-infrared and terahertz regions. Furthermore, the anisotropic response of the TaIrTe_4_ photodetector was quantified, showing that the degree of anisotropy escalates as the excitation wavelength approaches the Weyl node. Topological insulators have emerged as promising candidates for broadband photodetection due to their unique electronic structure. However, the practical applications of TIs are still in the early stages. Fabricating high-quality TI devices with well-defined surface states remains challenging. The coupling of TIs with other materials to realize new functionalities needs further optimization. Additionally, the stability and performance of TI-based devices under different environmental conditions require further research.

### 2.5. Black Phosphorus

Black phosphorus (BP), a stable allotrope of phosphorus in air, is a typical layered two-dimensional material. Each layer consists of corrugated atomic chains, where each atom engages in sp3 hybridization to form non-planar six-membered ring structures with three neighboring atoms. This material exhibits direct bandgap semiconductor characteristics, with the bandgap narrowing from 1.7 eV in monolayers to 0.3 eV in bulk forms as the number of layers increases [77,78]. Capitalizing on its narrow bandgap, BP has been widely applied in broadband photodetection.

**Figure 5:**
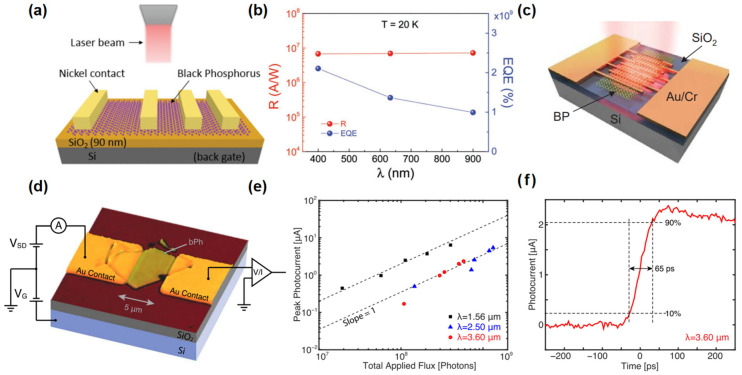
(**a**) Structure of the black phosphorus photodetector. (**b**) Responsivity and external quantum efficiency for different kinds of incident photons. Reproduced with permission from [79], Adv. Mater.; published by Wiley, 2016. (**c**) Structure of the black phosphorus photodetector. Reproduced with permission from [80], Nano Lett.; published by American Chemical Society, 2016. (**d**) Schematic diagram of the black phosphorus photodetector. (**e**) Peak photocurrent for all wavelengths plotted as a function of incident flux. For all measurements, a bias voltage of 200 mV and back gate of 0 V was used. (**f**) Photocurrent impulse response showing 65 ps rise time. Reproduced with permission from [82], 2D Mater.; published by IOP, 2016.

Huang et al. [79] developed a black phosphorus photodetector, as depicted in Figure 5a, which demonstrated high-performance detection across the 400–900 nm spectrum with a remarkable responsivity up to 10^6^ A/W level, as revealed in Figure 5b. Another BP photodetector, developed by Guo et al. [80] and shown in Figure 5c, achieved a responsivity of 82 A/W under 3.39 μm laser irradiation. Xu et al. [81] fabricated a BP photodetector that enables mid-infrared photodetection from 2.5 to 3.7 μm wavelengths. Ryan et al. [82] designed a photodetector illustrated in Figure 5d that leverages the light-guiding effect of black phosphorus for effective detection from near-infrared to mid-infrared (1.56–3.75 μm). This device exhibited an ultrafast response time of 65 ps when excited by a 3.6 μm laser irradiation, as given in Figure 5e,f. The performance of some broadband photodetectors based on pristine 2D materials is summarized in Table 1. Black phosphorus (BP) has attracted significant attention for broadband photodetection. However, BP also faces significant challenges related to environmental stability and carrier mobility. Additionally, achieving high carrier mobility in BP devices often requires precise control over the material’s thickness and doping levels. The scalability of BP and device fabrication also needs to be further explored.

### 2.6. Other Materials

Besides these 2D materials discussed earlier, several new 2D materials have gained tremendous interest, including transition metal monochalcogenides, 2D superalloys, and ternary bismuth telluride halides. For instance, Curreli et al. [83] fabricated the GaSe-based phototransistor using liquid-phase exfoliation, which had a high on/off ratio of ~10^3^, large responsivity of 13 A/W, and a fast response time of 35 ms. Similarly, Petrini et al. [84] prepared 2D BiTeI flakes using a gold-assisted exfoliation method, demonstrating remarkable nonlinear optical responses. These 2D materials have strong spin-orbit coupling, a tunable energy gap, and unique quantum behaviors, suggesting they should be highly attractive for next-generation photodetectors based on thermoelectric, piezoelectric, and nonlinear effects.

**Table 1:** Comparison of various 2D materials broadband photodetectors. NEP: Noise Equivalent Power. -: Not Applicable.

Device	Bias (V)	Range (nm)	Responsivity (A/W)	Detectivity (10^9^ Jones)	Rise Time (ms)	Fall Time (ms)	NEP [WHz^−1/2^]	EQE (%)	Ref.
SnSe	0.8	532–1064	2.14@532 nm	1.7@532 nm	70	69	7.4 × 10^−12^	499	[55]
Ta_2_NiSe_5_ nanosheets	1	405–2200	138.9@405 nm	8.4@405 nm	11.7 × 10^3^	15.9 × 10^3^	-	4.3 × 10^4^	[56]
Bi_2_O_2_Se thin film	−0.05	365–940	0.18@365 nm	1.2@470 nm	32	44	-	-	[57]
Sn-Pb perovskite films	0	350–1000	0.29@720 nm	1.6@720 nm	2 × 10^−3^	12.1 × 10^3^	1.06 × 10^−10^	-	[63]
CH_3_NH_3_PbBr_3_ crystal	1	355–1560	0.23@520 nm	143@520 nm	0.21 × 10^3^	0.93	-	55	[66]
(BA)_2_(MA)Sn_2_I_7_	1	365–1064	28.4@365 nm	23@365 nm	0.7 × 10^3^	1.2 × 10^3^	-	-	[64]
MAPbBr_3_ nanoplate	2	850–1450	5.04@520 nm	5370@520 nm	80 × 10^−3^	110 × 10^−3^	-	1200	[65]
Graphene	1	375 nm–118 μm	1@532 nm	-	0.16 × 10^3^	0.14 × 10^3^	-	-	[70]
Graphene	0	800–1600	0.025@1400 nm	-	0.2	0.2	4.4 × 10^−10^	-	[72]
TaIrTe_4_	0	532–10.6 μm	0.02@10.6 μm	0.18@10.6 μm	27 × 10^−3^	27 × 10^−3^	-	-	[76]
Black phosphorus	−1	400–900	10^6^@900 nm	-	-	-	-	1 × 10^9^	[79]
Black phosphorus	0	2.5 μm–3.7 μm	0.047@2.7 μm	-	-	-	-	2	[81]
Te nanosheets	3	261–405	65,000@261 nm	0.37@261 nm	2 × 10^3^	5 × 10^3^	-	2.2 × 10^6^	[85]
Sb_2_Se_3_ thin film	1	400–1200	3.37@1064 nm	100@1064 nm	73	69	2.95 × 10^−13^	369.3	[86]
SnTe nanosheets	1	254–4650	71.11@254 nm	-	0.21 × 10^3^	0.73 × 10^3^	-	-	[87]
GeTe nanofilm	0.8	600–900	100@850 nm	10,000@850 nm	-	-	-	~100	[88]
PdTe_2_	0.1	1 mm–7.5 mm	10@0.3 THz	-	1 × 10^−3^	2.2 × 10^−3^	2 × 10^−12^	-	[89]
PtTe_2_	−0.4	200–1650	0.406@980 nm	3620@980 nm	7.51 × 10^−3^	36.7 × 10^−3^	5.52 × 10^−15^	32.1	[90]
PdPs	1	254–1064	1180@532 nm	440@532 nm	1.4	1.2	-	-	[91]
InSiTe_3_ flakes	11	365–1310	0.07@365 nm	7.6@365 nm	5.45 × 10^−4^	5.76 × 10^−4^	-	-	[92]
Ga_2_In_4_S_9_ flakes	0	330–900	112@360 nm	225@360 nm	40	50	-	2.2 × 10^4^	[93]
AgSbTe_2_	1	405–980	0.024@405 nm	2@405 nm	0.49 × 10^3^	0.58 × 10^3^	-	-	[94]
MoS_2_	0	500–1100	1.358@950 nm	28@950 nm	0.71	0.66	4.5 × 10^−13^	476	[95]

## 3. Strategies for Enhancing Photodetection Performances

**Figure 6:**
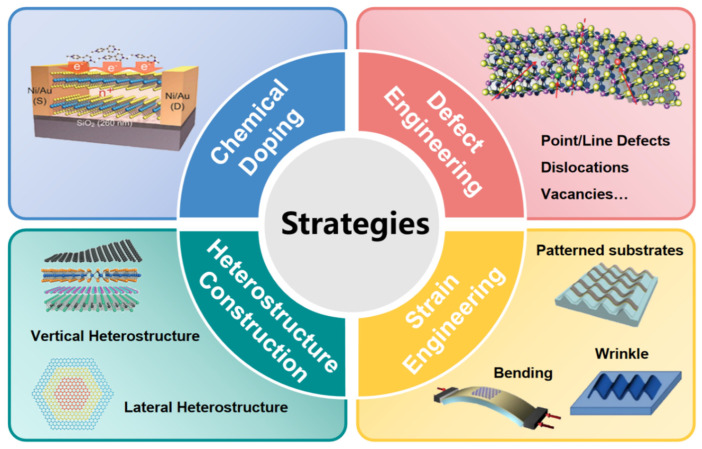
Schematic diagram of strategies for broadband photodetection optimization. Reproduced with permission from [96], Adv. Funct. Mater.; published by Wiley, 2021. Reproduced with permission from [97], Chem. Soc. Rev.; published by Royal Society of Chemistry, 2018. Reproduced with permission from [98], Superlattices Microstruct.; published by Elsevier, 2020. Reproduced with permission from [99], Infomat; published by Wiley, 2021.

As previously mentioned, devices based on pristine 2D materials for broadband photodetection have been extensively discussed. Despite notable progress, their performance has yet to fully meet the current requirements. Therefore, researchers have devoted considerable effort to developing innovative techniques and strategies to enhance the photodetection performance of these devices. Such methodologies include chemical doping to alter electronic properties, defect engineering to modulate carrier concentrations, heterostructure fabrication to create synergistic effects, and strain engineering to tune bandgaps and enhance light-matter interactions (Figure 6). These strategies are critical for enhancing the functionality and efficiency of 2D material-based broadband photodetectors, ensuring they can meet the demanding requirements of practical applications.

### 3.1. Chemical Doping

**Figure 7:**
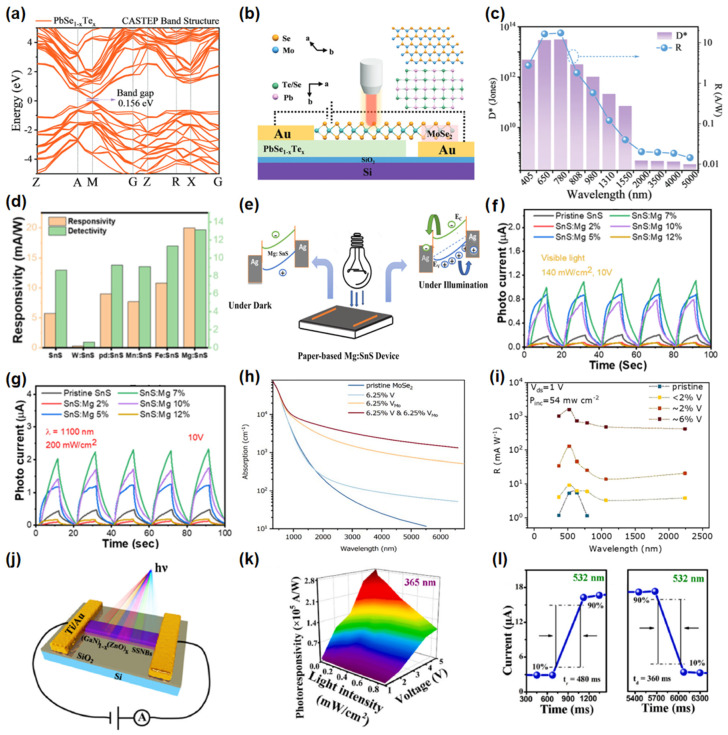
(**a**) DFT calculation band structures for PbSe_1-x_Te_x_. (**b**) The schematic diagram of the PbSe_1-_Te_x_/MoSe_2_ device and the atomic structure models of PbSe_1-x_Te_x_ and MoSe_2_. (**c**) Responsivity and detectivity of the PbSe_1-x_Te_x_/MoSe_2_ device from 405 to 5000 nm. Reproduced with permission from [102], Adv. Opt. Mater.; published by Wiley, 2023. (**d**) Responsivity and detectivity of different types of doped SnS photodetectors. (**e**) Schematic diagram and charge transfer mechanism for SnS/Mg. (**f**,**g**) I-t characteristics of SnS/Mg (2, 5, 7, 10, 12%) devices under visible and IR radiation. Reproduced with permission from [103], ACS Appl. Nano Mater.; published by American Chemical Society, 2024. (**h**) DFT-calculated absorption spectrum of Mo_16_Se_32_, V_1_Mo_15_Se_32_, Mo_15_Se_32_ and V_1_Mo_14_Se_32_. (**i**) Responsivity of photodetectors based on MoSe_2_ with different V compositions as a function of excitation wavelength. Reproduced with permission from [105], Appl. Surf. Sci.; published by Elsevier, 2021. (**j**) Optical images of the (GaN)_1-x_(ZnO)_x_ photodetector. (**k**) The responsivity of the (GaN)_1-x_(ZnO)_x_ photodetector under 365 nm irradiation. (**l**) The photoresponse time of the (GaN)_1-x_(ZnO)_x_ photodetector under 532 nm irradiation. Reproduced with permission from [106], Opt. Mater.; published by Elsevier, 2024.

Chemical doping fine-tunes the band structure by introducing impurity atoms, enabling broadband photodetection [100,101]. Peng et al. [102] synthesized a PbSe_0.5_Te_0.5_ atomic film through the incorporation of Te into PbSe, which resulted in a reduced bandgap and enhanced carrier mobility, leading to exceptional photo-response and broad-spectral detection capabilities as shown in Figure 7a. The doped PbSe_0.5_Te_0.5_ photodetector displays superior broadband photodetection performance (405–5000 nm), which is attributed to its narrow bandgap (Figure 7b,c). Parth et al. [103] synthesized a range of metal-doped SnS materials (Fe, Mg, Mn, Pd, W) via the hydrothermal method. Interestingly, Mg-doped SnS demonstrated the most pronounced response in both visible and near-infrared spectra among the various metal-doped SnS variants, as observed in Figure 7d,e. By modulating the energy band through Mg doping, the 7% Mg-doped SnS demonstrated excellent photo responsivity in the visible-infrared spectral, as seen in Figure 7f,g. Additionally, Parth et al. [104] effectively doped In into SnS to facilitate broad-spectrum photodetection ranging from 400 nm to 1100 nm by altering the bandgap from 1.44 eV to 2.08 eV. Through density functional theory calculations, Zhao et al. [105] found that the synergistic effect of vanadium substitution doping and molybdenum vacancies not only diminishes the bandgap but also enhances light absorption in monolayer MoSe_2_, as illustrated in Figure 7h. They successfully fabricated a photodetector based on monolayer MoSe_2_ with 6% V and Mo vacancies, which displayed a broadband spectral response from 365 nm to 2240 nm, and the responsivity reached 9.7 A/W and 2.8 mA/W at 520 nm and 2240 nm, respectively (Figure 7i). Our team [106] synthesized high-quality single-crystal (GaN)_1-x_(ZnO)_x_ nanobelts via the CVD method and fabricated a wide-spectral ultraviolet-visible photodetector, as illustrated in Figure 7j. The device demonstrates exceptional performance across the ultraviolet-to-visible light spectrum, attributed to its superior optical response and tunable bandgap. Specifically, under 365 nm ultraviolet irradiation, the photodetector achieves a responsivity of 2.8 × 10⁵ A/W, as shown in Figure 7k. Additionally, under 532 nm visible light irradiation, it exhibits a responsivity of 1.9 × 10⁴ A/W with a response time of 480 ms, as depicted in Figure 7l.

### 3.2. Defect Engineering

**Figure 8:**
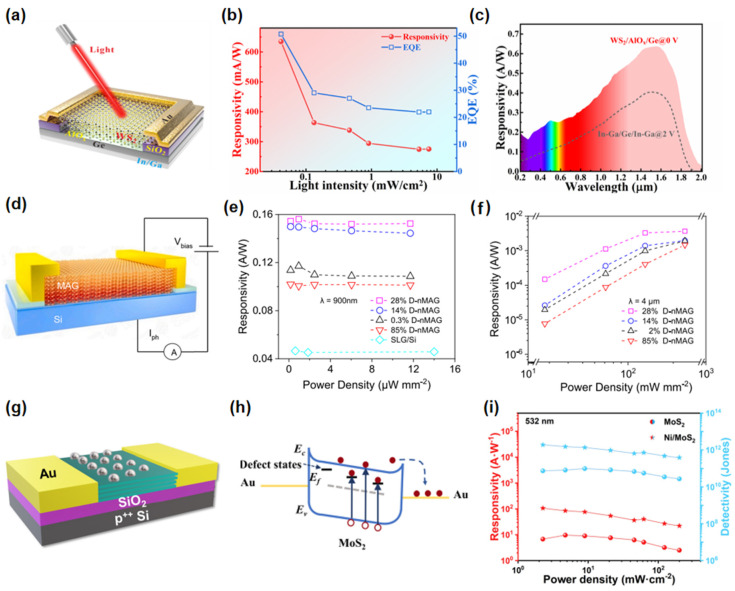
(**a**) Schematic diagram of the WS_2_/AlO_x_/Ge photodetector. (**b**) Responsivity and EQE values of the photodetector as a function of light intensity. (**c**) Spectral photoresponse of the WS_2_/AlO_x_/Ge photodetectors and pure Ge photodetectors. Under light illumination of 1550 nm. Reproduced with permission from [107], ACS Nano; published by American Chemical Society, 2021. (**d**) Schematic diagram of the D-nMAG/Si photodetector. (**e**) The responsivity of D-nMAG/Si as a function of laser power density at 900 nm. (**f**) The responsivity of D-nMAG/Si as a function of laser power density at 4 μm. Reproduced with permission from [109], Carbon; published by Elsevier, 2022. (**g**) Schematic illustration of the Ni/MoS_2_ photodetector. (**h**) Energy band diagram of the MoS_2_ photodetector under negative voltage. (**i**) Responsivity and detectivity of the MoS_2_ device and the Ni/MoS_2_ device under 532 nm illumination at 5 V. Reproduced with permission from [110], Research; published by AAAS, 2023.

Defects (e.g., vacancies, grain boundaries) in 2D materials critically modulate their electronic properties. By employing defect engineering strategies, these defects can be deliberately controlled and optimized, enhancing the performance of 2D material-based devices and overcoming the limitations imposed by their inherent bandgap. This approach has the potential to noticeably enhance the responsivity, response speed, and operational wavelength range of photodetectors, expanding their capabilities in optoelectronic applications. Wu et al. [107] developed WS_2_/Ge heterojunction photodetectors through defect engineering and interface passivation techniques, as shown in Figure 8a. The WS_2_/AlO_x_/Ge photodetector demonstrates exceptional performance attributed to a reduced bandgap resulting from defects. It exhibits a high responsivity of 634.5 mA/W, a detectivity of 4.3 × 10^11^ Jones, rapid response times, and an extensive spectral response ranging from 200 nm to 4.6 µm, as found in Figure 8b,c. Furthermore, this device exhibits remarkable MWIR imaging capabilities at room temperature. Xie et al. [108] achieved room-temperature THz photodetection in MoS_2_ by conducting bandgap engineering through the introduction of Mo^4+^ and S^2−^ vacancies. The generation and transport of excess charge carriers in the MoS_2_ sample are regulated by the vacancy concentration and resistivity in the THz electromagnetic radiation. A photo responsivity at 2.52 THz with 10 mA/W was achieved by balancing the fluctuation of carrier concentration and the scattering probability of charge carriers in the MoS_2.19_ sample.

In contrast to the bandgap modulation, defects also can act as efficient charge traps, achieving exceptional photocurrent gain in broadband photodetectors. Cao et al. [109] proposed a macro-assembled graphene nanofilm with precisely controlled defect states for wide-band infrared detection, investigating the correlation between the concentration of material defect states and detection performance. The device architecture is illustrated in Figure 8d. Defect states re-enter the conduction band (CB) and valence band (VB) within the D-nMAG trapped charge carriers for thermalization, resulting in enhanced photocurrent gain. Figure 8e presents the responsivity of various defect state concentrations of D-nMAG in the near-infrared (NIR) region at 900 nm, while Figure 8f displays the responsivity in the mid-infrared (MIR) region at 4 µm. Duan et al. [110] introduced a Ni/MoS_2_ photodetector, as illustrated in Figure 8g. This detector is modified with nickel nanoparticles on MoS_2_ that contain S vacancies, achieving excellent photodetection performance through defect engineering. The introduction of S vacancies enables effective light detection in the near-infrared range. Additionally, the photocatalytic effect of the nickel nanoparticles within the device reduces recombination rates and enhances hole transport. Figure 8h illustrates the schematic representation of the energy band structure under this configuration, thereby improving both the sensitivity and response speed of the photodetector. The MoS_2_ photodetector modified with nickel nanoparticles demonstrates responsivity of 106.21 A/W and 1.38 A/W under 532 nm and 980 nm light, with a detectivity of 1.9 × 10^12^ Jones and 8.9 × 10^9^ Jones, respectively, as revealed in Figure 8i.

### 3.3. Constructing Heterostructures

The two-dimensional materials’ lack of dangling bonds on their surfaces provides exceptional flexibility in integrating them with materials of other dimensions, including 0D, 1D, 2D, and 3D, without strict lattice matching requirements. This flexibility expands the possibilities for engineering diverse device architectures. By selectively combining complementary materials to form heterostructures, synergistic effects and functionalities surpassing those of individual components can be achieved, leading to significantly improved device performance and opening up new horizons for the design and novelty of photodetectors. Section 3.3 is divided into two parts, as Section 3.3.1 heterostructures are made of only 2D materials and Section 3.3.2 heterostructures combine 2D materials with other materials.

#### 3.3.1. Heterostructures Made of Only 2D Materials

**Figure 9:**
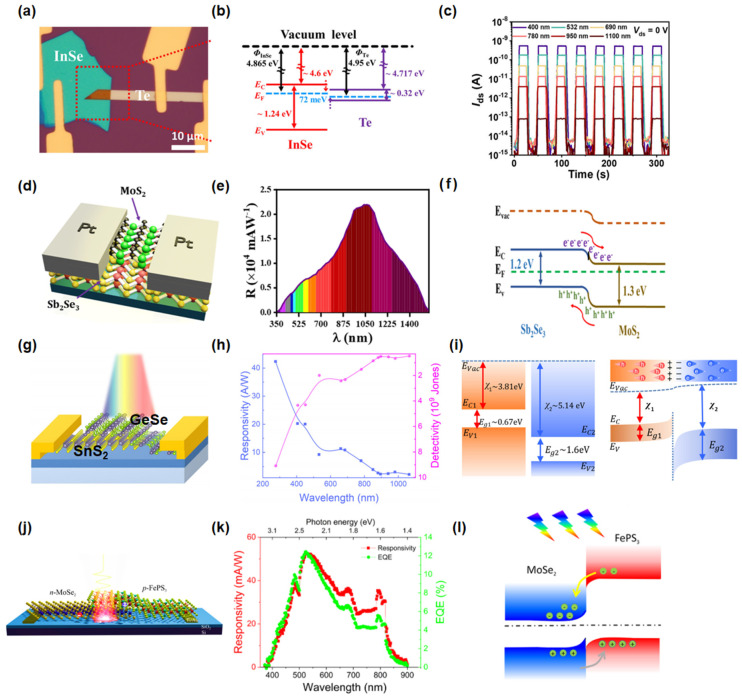
(**a**) Optical image of a typical InSe/Te heterostructure device. (**b**) Energy band profiles of InSe and Te. (**c**) Photoswitching behavior of the device under different light wavelengths. Reproduced with permission from [111], Laser Photonics Rev.; published by Wiley 2022 (**d**) Schematic of the MoS_2_/Sb_2_Se_3_ photodetector. (**e**) Spectral response of the MoS_2_/Sb_2_Se_3_ photodetector. (**f**) Band-diagram of the MoS_2_/Sb_2_Se_3_ photodetector. Reproduced with permission from [112], ACS Appl. Opt. Mater.; published by American Chemical Society, 2023. (**g**) Schematic image of the GeSe/SnS_2_ heterostructure photodetector. (**h**) The responsivity and detectivity curves irradiated under different wavelengths from 255 to 1064 nm. (**i**) The band alignment of the GeSe/SnS_2_ heterostructure before and after contact. Reproduced with permission from [113], Appl. Phys. Lett.; published by AIP, 2023. (**j**) Schematic diagram of the MoSe_2_/FePS_3_ photodetector. (**k**) Responsivity and EQE spectra of MoSe_2_/FePS_3_ photodetector measured at zero bias. (**l**) Straddling type-II configuration for multilayer MoSe_2_/FePS_3_. Reproduced with permission from [114], ACS Appl. Mater. Interfaces; published by American Chemical Society, 2022.

Combining different 2D materials can create heterojunctions with tailored band alignments that facilitate efficient charge separation and transport, leading to enhanced responsivity and detectivity. As shown in Figure 9a–c, our team [111] has developed a van der Waals heterostructure photodetector based on InSe/Te, which exhibits a broad spectral response from 400 to 1100 nm. The type-I band alignment allows the InSe component to selectively modulate the spectral response, while the Te layer efficiently collects photogenerated holes, suppressing carrier recombination and enhancing device performance. This heterostructure achieves an optical on/off ratio of up to 10^5^ A/W and a detectivity of 1.77 × 10^11^ Jones. Vashishtha et al. [112] constructed a MoS_2_/Sb_2_Se_3_ heterojunction photodetector (Figure 9d), which forms a type-II band alignment. This alignment creates a small barrier potential at the interface, minimizing the bending effect in the energy band diagram and increasing the number of accessible states for charge carriers. As a result, the device demonstrates high responsivity and detectivity across the visible to infrared spectral range (Figure 9e,f). Similarly, the GeSe/SnS_2_ van der Waals heterostructure exhibits exceptional photodetection capabilities, showing a broad spectral response from ultraviolet to near-infrared wavelengths (255–1920 nm), as shown in Figure 9g–i [113]. The type-III band alignment of GeSe/SnS_2_ creates an internal electric field that facilitates efficient interlayer charge transfer, leading to a high responsivity of 50.7 A/W and rapid response times (2.1 ms). Notably, certain heterostructures exhibit self-powered operation due to built-in electric fields at the interface. Duan et al. [114] fabricated a MoSe_2_/FePS₃ van der Waals heterojunction photodetector (Figure 9j–l), where the type-II band alignment generates an internal electric field, enabling self-powered detection across 350–900 nm with a responsivity of 52 mA/W at 522 nm.

#### 3.3.2. Heterostructures Combining 2D Materials with Other Materials

**Figure 10:**
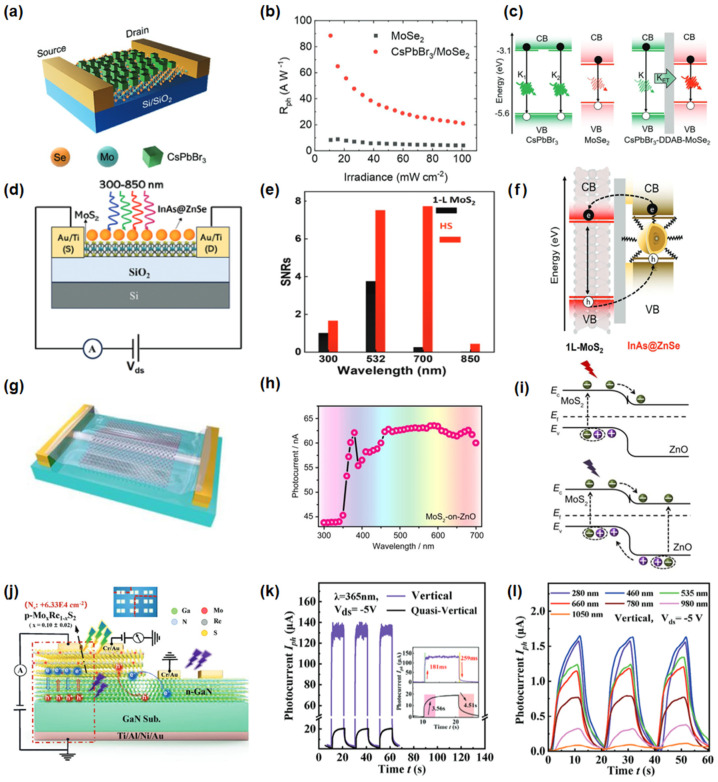
(**a**) Schematic diagram of CsPbBr_3_/MoSe_2_ photodetectors. (**b**) Photoresponsivity versus the irradiance curves of the CsPbBr_3_/MoSe_2_ heterojunction. (**c**) Schematic model depicting the major photoinduced processes in the single components (**left**) of CsPbBr_3_ (green) and MoSe_2_ (red) and the heterojunction (**right**). Reproduced with permission from [116], Adv. Opt. Mater.; published by Wiley, 2022. (**d**) Schematic diagram of the InAs@ZnSe/MoS_2_ photodetectors. (**e**) The signal-to-noise ratio for pristine 1L-MoS_2_ and InAs@ZnSe/MoS_2_ heterojunction under the same conditions is illustrated. (**f**) The schematic illustration depicts the process of charge transfer between 1L-MoS_2_ and InAs@ZnSe. Reproduced with permission from [117], Adv. Funct. Mater.; published by Wiley, 2024. (**g**) Schematic diagram of the MoS_2_-on-ZnO heterojunction photodetectors. (**h**) Photocurrent curve of the MoS_2_-on-ZnO heterojunction photodetectors as a dependence of wavelength. (**i**) Schematic diagrams of the band energy structure of the MoS_2_-on-ZnO heterojunction under UV and visible light. Reproduced with permission from [118], Tungsten; published by Springer,2023. (**j**) Photodetector based on the quasi-vertical and vertical heterostructure of p-Mo_x_Re_1-x_S_2_/GaN. (**k**) Rise and fall times of the high-resolution time-resolved photocurrent response of the Vertical and Quasi-vertical photodetector. (**l**) The time-resolved photo response of the Vertical p-Mo_x_Re_1-x_S_2_/GaN heterostructure photodetector under different wavelength excitation at −5 V bias. Reproduced with permission from [119], Adv. Opt. Mater.; published by Wiley, 2024.

Heterostructures formed by integrating 2D materials with 0D, 1D, or 3D systems expand the possibilities for advanced photodetectors. These hybrid architectures also enable unique functionalities such as self-powered operation and wavelength-dependent photoresponse modulation [115]. As shown in Figure 10a–c, Asaithambi et al. [116] developed a 0D/2D heterojunction using CsPbBr_3_ nanoparticles and monolayer MoSe_2_. The efficient energy transfer at the interface generates a significant number of free charge carriers, enabling the device to achieve a high responsivity of 88 A/W. Afterwards, Asaithambi et al. [117] further constructed a 0D/2D heterostructure integrating InAs@ZnSe nanoparticles with monolayer MoS_2_ (Figure 10d–f). The enhanced charge transfer dynamics at the interface not only boost the responsivity to 10^3^ A/W but also exhibit a broadband optical response from 300 to 850 nm and an exceptional specific detectivity of 10^11^ Jones. These results suggest that interface engineering should be an effective way to improve the photoresponse performances of broadband photodetectors. Our team [118] constructed a MoS_2_-on-ZnO (1D/2D) vertical heterojunction photodetector, as illustrated in Figure 10g–i. The ZnO nanowires provide a fast carrier transport pathway, while MoS_2_ responds to visible light. The device achieves 273 A/W responsivity under UV light and 74 A/W under visible light, with response times <24 ms. Liu et al. [119] designed a fully vertical 2D/3D van der Waals stacked p-Mo_x_Re_1-x_S_2_/n-GaN heterojunction photodetector. The device demonstrates high responsivity (888.69 A/W), detectivity (6.13 × 10^14^ Jones), and broad spectral response (UV to NIR), as presented in Figure 10j–l.

**Figure 11:**
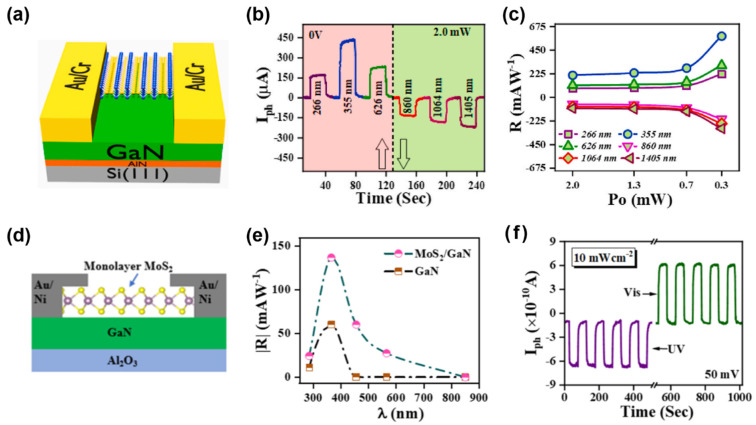
(**a**) Schematics of the Bi_2_Se_3_/GaN photodetector. (**b**) Wavelength-dependent I − T under various illumination wavelengths. (**c**) The wavelength-correlated power-dependent characteristics of responsivity. Reproduced with permission from [120], Mater. Sci. Semicond. Process.; published by Elsevier, 2024. (**d**) Schematic of the MoS_2_/GaN photodetector. (**e**) Spectral response at 10 mW cm^−2^ and for the MoS_2_/GaN heterostructure compared with the bare GaN. (**f**) I−T measurement at a fixed bias and different illumination wavelength. Reproduced with permission from [121], ACS Appl. Mater. Interfaces; published by American Chemical Society, 2025.

Furthermore, as shown in Figure 11a–c, Vashishtha et al. [120] reported an Sb_2_Se₃/GaN heterojunction photodetector with a self-powered ultra-broadband response (250–1250 nm) and a 153% enhancement in responsivity through wavelength-modulated carrier dynamics. The device exhibits a forward responsivity of 0.58 A/W at 355 nm (UV) and a negative responsivity of −0.3 A/W at 1405 nm (IR). Similarly, Walia et al. [121] developed a MoS_2_/GaN heterostructure photodetector (Figure 11d) that operates in a self-powered mode from 285 to 850 nm, achieving a peak responsivity of 0.63 A/W at 365 nm. Remarkably, the device generates a negative photocurrent under UV light and a positive photocurrent under visible illumination (Figure 11e,f), highlighting significant potential applications in optoelectronics, neuromorphic computing, and sensing technologies.

### 3.4. Strain Engineering

The remarkable mechanical flexibility of 2D materials allows for the application of substantial strain, which in turn can significantly modify the electronic, optical, and transport properties of these materials. This strain-induced modulation of properties opens up new possibilities for optoelectronic device applications, paving the way for the development of innovative and high-performance devices [122,123,124]. Strain engineering enhances the performance of wide-spectral photodetectors by adjusting the band structure, light absorption, and charge carrier mobility of the materials [99,125,126].

**Figure 12:**
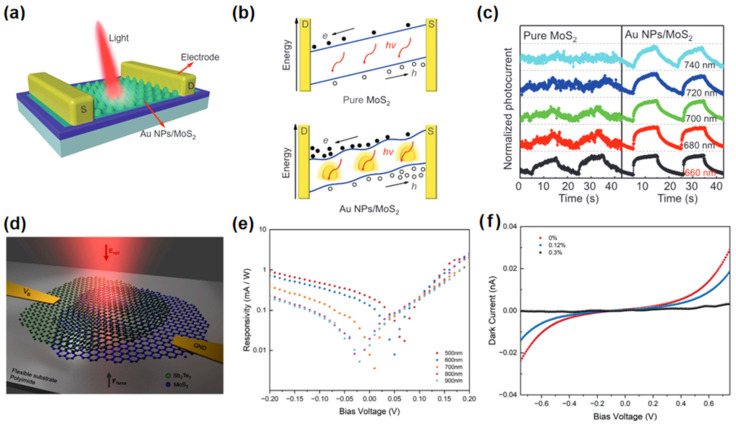
(**a**) Schematics of the strain-plasmonic coupled photodetector. (**b**) Schematic energy band diagram of the photodetection mechanism. (**c**) Time-dependent photo-response of pure MoS_2_ and Au NPs/MoS_2_ photodetectors under 660 nm, 680, 700, 720, and 740 nm light illumination. Reproduced with permission from [127], Small; published by Wiley, 2022. (**d**) Schematic of the Sb_2_Te_3_/MoS_2_ photodetector. (**e**) The measured responsivity at different wavelengths (500 nm, 600 nm, 700 nm, 800 nm, and 900 nm). (**f**) The I–V characteristic of the Sb_2_Te_3_/MoS_2_ photodetector measured under varying strains. Reproduced with permission from [128], Nanomaterials; published by MDPI, 2023.

Lu et al. [127] designed a strain-plasmonic coupled MoS_2_ photodetector by transferring a monolayer of MoS_2_ onto a pre-fabricated array of gold nanoparticles, as shown in Figure 12a. This design enables significant biaxial tensile strain, which narrows MoS_2_’s wide bandgap and enhances light absorption of MoS_2_ due to the gold nanoparticles. Figure 12c shows the strain-plasmonic coupled photodetector exhibited an expanded detection range of 60 nm and a remarkable enhancement in signal-to-noise ratio by 650%, optimizing both detection range and responsivity. Wang et al. [128] demonstrated a MoS_2_/Sb_2_Te_3_ photodetector (Figure 12d) that exhibited substantial tunability under compressive strain of up to 0.3%. The strain at the heterojunction interface influenced the bandgap of MoS_2_/Sb_2_Te_3_, altering the heterojunction band structure and modulating the detector’s optical absorption characteristics. Figure 12e illustrates the wide-spectral response range of the device, spanning from 500 to 900 nm. Under strain, the bandgap and resistance increase while the dark current decreases, reducing responsivity and enhancing the photodetector’s versatility for practical applications (Figure 12f).

**Figure 13:**
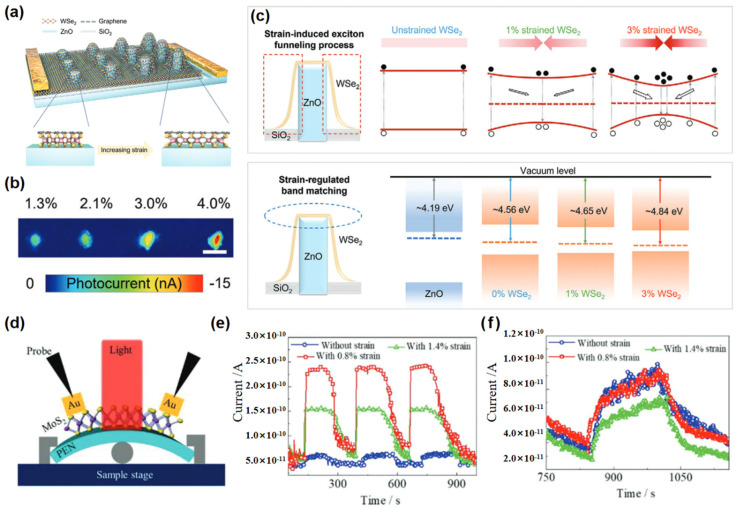
(**a**) Schematic diagram of the heterostructure with gradient strain modulation. (**b**) SPCM of the vdWs heterostructure at zero bias voltage. (**c**) The mechanism explanation of the strain-enhanced optoelectronic performance of the ZnO/WSe_2_ heterojunction. Reproduced with permission from [129], Adv. Funct. Mater.; published by Wiley, 2024. (**d**) Schematic diagram for photoelectric performance measurement under strain. (**e**) Time dependence of source-drain current of the photodetector during the light switching in armchair direction. (**f**) Time dependence of the source-drain current of the photodetector during the light switching in a zigzag direction. Reproduced with permission from [130], Adv. Electron. Mater.; published by Wiley, 2019.

In addition, Zeng et al. [129] introduced a gradient strain modulation strategy in 2D materials, significantly enhancing the photodetection performance of a ZnO/WSe_2_/graphene photodetector (Figure 13a). In contrast to conventional photodetectors where all components experience uniform strain, the biaxial tensile strain in WSe_2_ can be finely tuned by adjusting the height of ZnO nanorods, with minimal impact on ZnO. As the strain modulation increased from 1.3% to 4.0%, the EQE of the photodetector rose from 11.4% to 35.3% (Figure 13b). The primary factors contributing to this enhancement in photodetection capability are illustrated in Figure 13c. The gradient strain develops a built-in electric field across various strained regions within WSe_2_, and the high exciton binding energy of WSe_2_ directs photo-generated electron-hole pairs towards areas of concentrated strain, increasing charge carrier density at the ZnO-WSe_2_ interface and facilitating charge separation. Additionally, the decrease in WSe_2_’s Fermi level with increasing strain increases the Fermi level difference between ZnO and WSe_2_, enhancing the built-in potential at the interface and driving charge separation. Li et al. [130] developed a photodetector (Figure 13d) to investigate the influence of strain on atomic arrangement across various orientations. Figure 13e,f show that under a bending strain of 0.8%, the self-powered photoelectric current is significantly greater when the electrode is aligned perpendicular to the armchair direction compared to the zigzag orientation, demonstrating the significant impact of strain on device performance. A summary of the performance of some broadband photodetectors in recent years is provided in Table 2.

## 4. Outlook and Conclusions

This review article has comprehensively summarized the recent advancements in 2D material-based broadband photodetection, including an in-depth discussion of their intrinsic optoelectronic properties, enhancing strategies, and challenges. It provides critical insights into the strengths and weaknesses of each material and its corresponding devices. Although 2D materials have obtained some encouraging achievements in broadband photodetection, three critical challenges hinder their practical applications: controllability in synthesis, batch-to-batch reproducibility, and long-term stability. Environmental factors such as temperature, strain, and humidity severely impact device uniformity and durability. For instance, temperature fluctuations induce lattice expansion and alter carrier scattering rates, while humidity accelerates decomposition in materials like perovskites. Strain engineering modulates the electronic properties of 2D materials but requires precise control. Strategies such as efficient thermal management and the development of materials with lower temperature coefficients can mitigate the effects of temperature variations. Encapsulation techniques protect devices from humidity-induced degradation. Standardized fabrication processes are essential for reducing variability. Moreover, surface passivation techniques improve stability by reducing defect density and protecting materials from environmental degradation. Addressing these factors is critical to the development of 2D materials broadband photodetectors with high controllability, reproducibility, and stability.

Future research should focus on material innovation, advanced strategies, and scalable fabrication techniques. Firstly, the development of narrow-gap materials is urgently desired for broadband photodetection because they have tunable bandgaps, good chemical stability, high carrier mobility, and low dark current at the same time. The researchers should explore more novel 2D materials suitable for broadband detection using a combination of theoretical calculations and experiments. Secondly, mastering more novel strategies for enhancing photodetection performances of 2D materials is very crucial, which can simultaneously have high responsivity and fast response time. They usually consist of four strategies based on the reported results, in which the use of a novel heterojunction or the structure design or optimization of a 2D material-based photodetector should be more effective. Through a combination of broadband light absorption, advanced environmental sensing and energy conversion, the integration of 2D materials into photoelectrochemical (PEC) devices may offer an interesting research field for future multifunctional detectors [134,135]. Thirdly, large-scale integration of 2D materials is another challenge, including scalable production technique, compatibility with traditional plane technology, device uniformity, and long-term work stability in various conditions. Additionally, the newly developed synthesis methods, such as gold-assisted mechanical exfoliation and liquid-phase exfoliation, may open new opportunities for future wearable electronic devices [136,137,138,139].

In conclusion, 2D materials should have particular advantages for broadband photodetection, which need to be paid much attention to advocate their practical applications in next-generation optoelectronic information technology.

## Figures and Tables

**Table 2:** The comparable table of the working performance of some broadband photodetectors in recent years.

Device	Strategies	Range (nm)	Responsivity (A/W)	Enhancement	Ref.
PbSe_0.5_Te_0.5_	Chemical Doping	405–5000	17.5@780 nm	-	[102]
Mg-doped SnS	Chemical Doping	400–1100	0.052@470 nm	344%	[103]
MoSe_2_ with 6% V	Chemical Doping	365–2240	9.7@520 nm	625%	[105]
WS_2_/AlO_x_/Ge	Defect Engineering	200–4600	0.63@1550 nm	150%	[107]
D-nMAG	Defect Engineering	900–4000	0.15@900 nm	385%	[109]
CdS_x_Se_1-x_/Te	Heterostructure	355–800	435@vis	446%	[131]
MoSe_2_/FePS_3_	Heterostructure	350–900	0.052@522 nm	144%	[114]
p-Mo_x_Re_1-x_S_2_/GaN	Heterostructure	280–1050	888.69@365 nm	352%	[119]
Sb_2_Se_3_/GaN	Heterostructure	250–1250	1.2@355 nm	153%	[132]
SnSe/GaN	Heterostructure	250–1250	128@355 nm	-	[133]
Bi_2_Se_3_/GaN	Heterostructure	266–1405	0.58@355 nm	148%	[120]
MoS_2_	Strain Engineering	660–740	660@418 nm	400%	[127]
MoS_2_/Sb_2_Te_3_	Strain Engineering	500–900	0.001@600 nm	-	[128]

## Data Availability

Data is contained within the article.
